# Potential of interleukin-7 in sepsis as a biomarker and therapeutic agent: a narrative review

**DOI:** 10.3389/fmed.2025.1649049

**Published:** 2025-10-13

**Authors:** Tong Zhang, Han Liu, Yi-fu Tie, Tian-wei Meng, Qun Liang

**Affiliations:** ^1^Heilongjiang University of Chinese Medicine, Harbin, China; ^2^Department of Epidemiology and Public Health, University College London, London, United Kingdom; ^3^Ordos Hospital of Traditional Chinese Medicine, Ordos, China; ^4^Department of Critical Care Medicine, The First Affiliated Hospital, Heilongjiang University of Chinese Medicine, Harbin, China

**Keywords:** sepsis, IL-7, septic shock, immune dysfunction, interleukin-7

## Abstract

**Objective:**

This study seeks to examine the dynamics of endogenous interleukin-7 (IL-7) and its associated regulatory factors in sepsis, and to elucidate the mechanisms by which exogenous IL-7 may confer therapeutic benefits. The ultimate objective is to evaluate its dual potential as a clinical biomarker and as a novel therapeutic agent.

**Method:**

We searched PubMed, Embase, and Web of Science from inception to April 24, 2025, using terms such as Interleukin-7, CD127, CYT107, interleukin-7 receptor, sepsis, septic shock, and lymphopenia.

**Results:**

In sepsis, endogenous IL-7 levels rise from a low baseline and may remain elevated for a prolonged period. Exogenous IL-7 can enhance immune function, regulate inflammation, and exert anti-apoptotic effects. Endogenous IL-7 levels may represent a potential prognostic indicator in sepsis. Exogenous IL-7 may modulate immune function in patients with clinical sepsis but does not reduce mortality.

**Conclusion:**

Endogenous IL-7 is closely associated with sepsis, whereas exogenous IL-7 shows promise for aiding the recovery of patients with sepsis, although further research is required.

## 1 Introduction

Sepsis is a serious disease associated with high morbidity, characterized by severe organ dysfunction induced by infection (1–4). Sepsis troubled 48.9 million people globally, causing 11.0 million deaths and a major socioeconomic burden (5). The prevalence was 22.4%, with 20.9% in low- and lower-middle-income countries. Kidney injury is a common complication, occurring in approximately 18% of cases (6, 7). Sepsis typically arises from infections, sterile inflammation, autoimmune diseases, and cancers (8). Smoking, alcohol consumption, and vitamin D deficiency are common risk factors for sepsis (8). Recently, studies have comprehensively examined the rehospitalization of sepsis patients, identifying aging and male gender as high-risk factors (9). Sepsis can be divided into adult and pediatric forms based on age (3, 4). Additionally, it can be classified by complications, such as respiratory, brain, and kidney sepsis, among others (10). In the affected organs, the primary pathological features include endothelial changes, high glycolysis, microcirculatory dysfunction, barrier damage, and immune system dysfunction. At the molecular level, the pathological mechanisms involve inflammation, oxidative stress, complement activation, and metabolic dysfunction (8, 11–14). Among these changes, dysfunction of the immune system represents a key pathological alteration during the course of sepsis.

Early screening for sepsis is crucial for reducing mortality. Recommended methods include using SOFA in combination with SIRS or NEWS as a single screening tool (15). However, the prognosis of sepsis is difficult to predict owing to the heterogeneity of the condition at the individual patient level (16). In the management of sepsis, fluid resuscitation and antibiotics remain the cornerstone of therapy, with corticosteroids administered as an adjunct in selected cases (17, 18). However, the bacterial drug resistance is a significant challenge. Fluid therapy is merely a basic approach to alleviate symptoms and reduce pathogen loads. Moreover, corticosteroids may exacerbate immune dysfunction (19, 20). Therefore, additional prognostic markers and therapeutic agents need to be explored.

Interleukin-7 (IL-7), a 25 kDa protein, could be expressed in a general tissue but the lymph nodes, liver, lung, and skin are high. In the cellular sources, the epithelial and endothelial cells are identified as sources (21). The IL-7 receptor (IL-7R) consists of *α*-subunit (CD127) and *γ*-chain, and is expressed in CD4^+^ and CD8^+^ T cells (21–23).

Several studies have demonstrated a strong association between IL-7, IL-7R, and immune function. HIV patients typically exhibit decreased IL-7R levels and impaired endogenous IL-7 function. In healthy individuals, endogenous IL-7 is present at significant concentrations and can increase following antiretroviral therapy in HIV patients (24, 25). These results suggest that endogenous IL-7 and IL-7R may hold value as prognostic markers in sepsis. In recent years, immunotherapy has attracted growing attention (26, 27) and exogenous IL-7 has shown potential as an immunotherapeutic agent. Exogenous IL-7 can enhance lymphocyte function through various pathways (24, 25, 28–35). COVID-19 has recently become a well-known virus. The administration of exogenous IL-7 could potentially benefit patients suffering from COVID-19 and other viral infections (36–41). Sepsis patients often have compromised immune function, and several studies have shown that IL-7 can regulate immune by enhancing T cell activity (42–44). Moreover, IL-7R appears to be associated with sepsis mortality (45). The potential of IL-7 in treating sepsis is promising.

## 2 Methods

We searched PubMed, Embase, and WOS from inception to April 24, 2025, for a comprehensive review. We used a combination of search terms, including “Interleukin-7,” “CD127,” “CYT107,” “Interleukin-7 receptor,” “sepsis,” “septic shock,” and “lymphopenia.” These terms were carefully selected to capture a wide range of relevant studies and articles related to IL-7 and its role in sepsis. We included animal experiments, *ex vivo* preclinical trials, and clinical trials, without language restrictions, to explore changes in endogenous IL-7, IL-7R, and related factors in sepsis, as well as the role of exogenous IL-7 in this condition, with the aim of providing greater reliability for the clinical application of IL-7 and IL-7R in sepsis. Articles excluded from the analysis comprised case reports, reviews, and studies in which sepsis was not the primary disease. No methods were employed to assess either the robustness of the data synthesis or the certainty of the evidence.

## 3 Results

### 3.1 The change of IL-7 and relevant factors in sepsis

**Figure 1:**
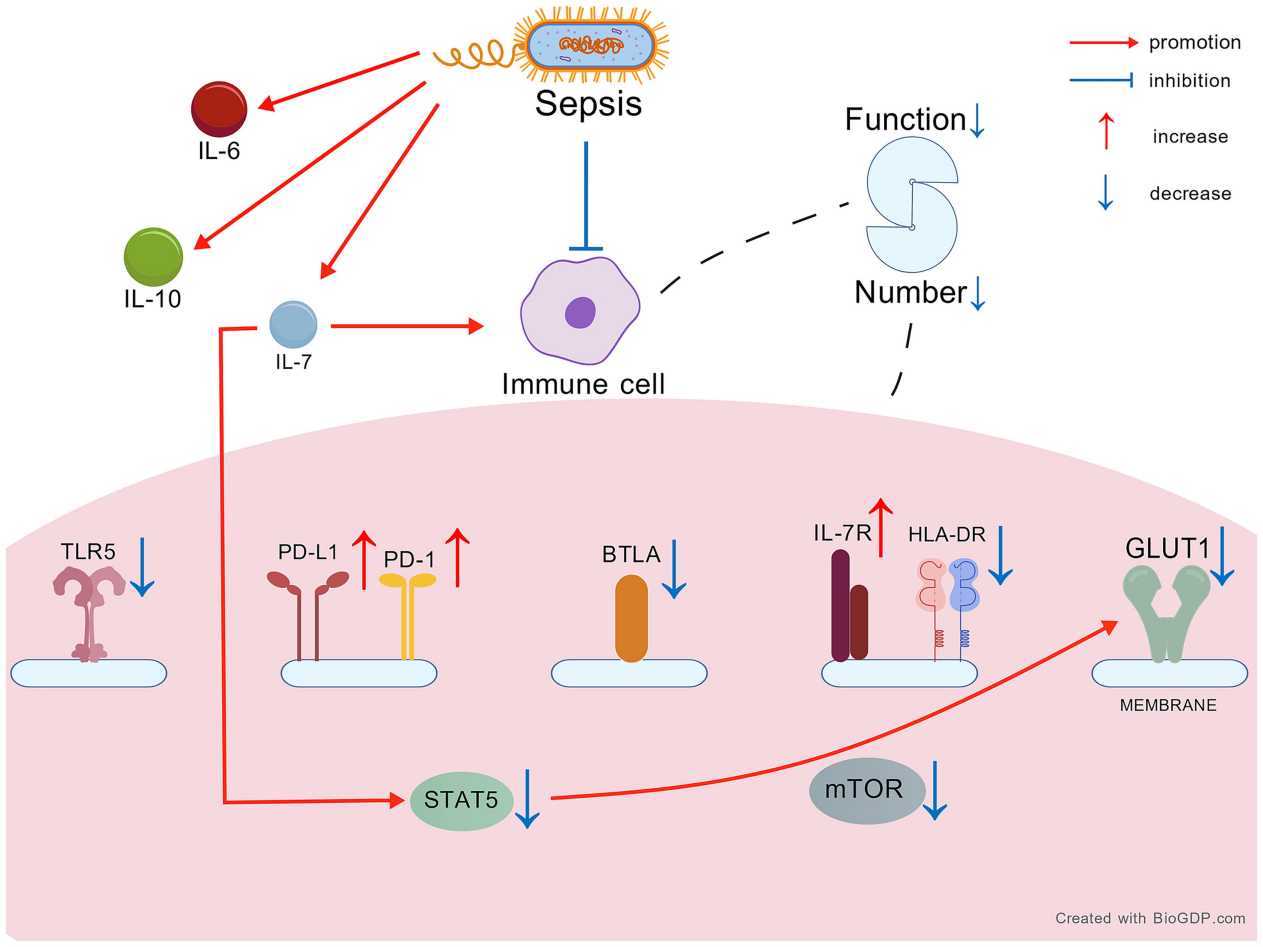
The change of IL-7 and relevant factors in sepsis. Created with BioGDP.com (130). IL-7, interleukin-7; IL-7R, IL-7 receptor; STAT5, signal transducers and activators of transcription 5; HLA-DR, human leukocyte antigen DR; PD-1, programmed cell death 1; PD-L1, programmed cell death ligand 1; TLR5, Toll-like receptor 5; BTLA, B- and T lymphocyte attenuator; GLUT1, glucose transporter 1; mTOR, mammalian target of rapamycin; IL-6, interleukin-6; IL-10, interleukin-10.

We included 16 studies examining changes in endogenous IL-7 and related factors (Table 1). When sepsis occurs, the immune system is often suppressed. Sepsis patients exhibit substantial alterations in IL-7, IL-7R, and associated factors (Figure 1).

**Table 1:** The change of IL-7 and relevant factors in sepsis.

References	Object	Model	Control	Source	Variation
(46)	IL-7	Sepsis patients	Health volunteer	plasma	↑
	IL-7R	Sepsis patients	Health volunteer	CD4^+^ and CD8^+^ T cells	—
	IL-7R	Sepsis patients	Health volunteer	Plasma	↑
(48)	IL-7 mRNA	Severe sepsis patients	Sepsis patients	Plasma	↓
(47)	IL-7	Sepsis patients	Health volunteer	Serum	↓
	IL-7	Severe sepsis patients	Sepsis patients	Serum	↑
(64)	CD4^+^ T cells	Sepsis patients	Health volunteer	Plasma	↓
	CD4^+^ FOXP3^+^ T cells	Sepsis patients	Health volunteer	Plasma	↑
	HLA-DR	Sepsis patients	Health volunteer	Monocytes	↓
	pSTAT5	Sepsis patients	Health volunteer	CD4^+^ T cells	↓
	pSTAT5	Sepsis death	Sepsis survivor	CD4^+^ T cells	↓
(82)	CD25^+^ CD127^−^ T cells	Sepsis survivor	Health volunteer	Plasma	—
	PD-1	Sepsis survivor	Health volunteer	CD4^+^ T cells	↓
	PD-1	Sepsis survivor	Health volunteer	CD8^+^ T cells	—
	PD-L1	Sepsis survivor	Health volunteer	Monocytes	—
	TLR5	Sepsis survivor	Health volunteer	Monocytes	↓
	BTLA	Sepsis survivor	Health volunteer	CD4^+^ T cells	↑
(57)	Metabolic status	Sepsis patients	Health volunteer	Lymphocytes	↓
	Aerobic glycolysis	Sepsis patients	Health volunteer	Lymphocytes	↓
	GLUT1	Sepsis patients	Health volunteer	CD4^+^ T cells	↓
	mTOR	Sepsis patients	Health volunteer	Lymphocytes	↓
(85)	IL-6	Sepsis patients	Health volunteer	Plasma	↑
	IL-10	Sepsis patients	Health volunteer	Plasma	↑
(65)	ILCs	Sepsis patients	Health volunteer	Plasma	↓
	ILC1	Sepsis patients	Health volunteer	Plasma	↓
	ILC3	Sepsis patients	Health volunteer	Plasma	↓
	HLA-DR	Sepsis patients	Health volunteer	ILCs	↓
(53)	IL-7	Sepsis survivor	Sepsis patients	Plasma	↑
	IL-7	Sepsis survivor after 1 year	Health volunteer	Plasma	—
(71)	Lymphocytes	Sepsis patients of multidrug resistant bacterial	Critically-ill non-septic patients	Plasma	↓
	Monocytes	Sepsis patients of multidrug resistant bacterial	Critically-ill non-septic patients	Plasma	↓
	PD-1	Sepsis patients of multidrug resistant bacterial	Critically-ill non-septic patients	CD4^+^ and CD8^+^ T cells	↑
	PD-L1	Sepsis patients of multidrug resistant bacterial	Critically-ill non-septic patients	Monocytes	↑
	HLA-DR	Sepsis patients of multidrug resistant bacterial	Critically-ill non-septic patients	Monocytes	↓
(131)	IL-7	Sepsis death with AD	AD death without systemic infection	Autopsy-acquired brain tissue	↓
(54)	IL-7R mRNA	Sepsis patients	Health volunteer	Plasma	↑
	IL-7R mRNA	Sepsis survivor	Sepsis death	Plasma	↑
(80)	CD127-PD-1^+^ T cells	Sepsis survivor	Health volunteer	Plasma	↑
	HLA-DR	Sepsis patients	Health volunteer	CD127^low^ PD-1^high^ T cells	↑
(52)	IL-7	Sepsis survivor of children	Health volunteer	Plasma after T cell activation	↑
(56)	HLA-DR	Sepsis patients and COVID-19 patients	Health volunteer	Monocytes	↓
	CD4^+^ T cells	Sepsis patients and COVID-19 patients	Health volunteer	Plasma	↓
	secretion and proliferation function of T cells	Sepsis patients and COVID-19 patients	Health volunteer	Plasma	↓

IL-7, interleukin-7; IL-7R, IL-7 receptor; CD127, IL-7 by the α-subunit; STAT5, signal transducers and activators of transcription 5; HLA-DR, human leukocyte antigen DR; ILC, innate lymphoid cell; PD-1, programmed cell death 1; PD-L1, programmed cell death ligand 1; TLR5, Toll-like receptor 5; BTLA, B- and T lymphocyte attenuator; GLUT1, glucose transporter 1; mTOR, mammalian target of rapamycin; IL-6, interleukin-6; IL-10, interleukin-10; ↑, increase; ↓, decrease; —, maintain.

#### 3.1.1 Elevated IL-7 and IL-7R in the plasma of sepsis patients from a low baseline

Compared to healthy individuals, IL-7 in the plasma of sepsis patients slightly increase within 1–4 days (46). However, IL-7 levels are relatively low compared with those in healthy individuals prior to any therapeutic intervention. Patients with severe sepsis exhibit higher IL-7 concentrations than those with uncomplicated sepsis, yet these levels remain lower than in healthy controls before treatment. Interestingly, patients with severe sepsis display lower blood IL-7 mRNA expression than individuals with less severe disease (47, 48). Moreover, individuals with Alzheimer’s disease who die from sepsis have lower IL-7 levels in the brain (49–51). During sepsis, IL-7R levels increase, particularly on CD4^+^ and CD8^+^ T cells, and remain elevated for 1–4 days (46). In patients with sepsis, IL-7R mRNA levels are higher than in healthy individuals. Overall, IL-7 exhibits marked fluctuations during sepsis. Its plasma concentration rises sharply within the first 1–4 days and remains elevated from a low baseline, compared with healthy controls, at the initiation of conventional treatment; a similar pattern is observed for IL-7R.

#### 3.1.2 The higher levels of plasma IL-7 and IL-7R in sepsis survivors than health

In sepsis survivors, IL-7 levels can rise and remain comparable to those of healthy individuals for up to one year. Similarly, pediatric sepsis survivors often have higher IL-7 levels compared to healthy children (52, 53). Additionally, plasma IL-7R concentrations are higher in sepsis survivors than in those who succumb to the condition, at least 2 days after onset (54). This suggests that, although patients with sepsis have impaired IL-7 function, they are still capable of secreting higher amounts of IL-7, likely as a result of epithelial and endothelial cell activation (54, 55). Additionally, immune dysfunction in sepsis may impair the clearance of IL-7, thereby contributing to its sustained levels. Survival in patients with sepsis may be associated with higher numbers of active lymphocytes and elevated IL-7 concentrations compared with healthy individuals.

#### 3.1.3 The change of relevant factors in sepsis

##### 3.1.3.1 The metabolic and proliferation disorder of immune system

Aerobic glycolysis is a crucial pathway for the growth and differentiation of lymphocytes (55). In sepsis patients, lymphocyte metabolism and aerobic glycolysis are impaired, and T cell secretion and proliferation functions are diminished (56, 57), indicating lymphocyte dysfunction. IL-7 can activate signal transducers and activators of transcription 5 (STAT5) to promote lymphocyte function (58–63). In sepsis patients, p-STAT5 are reduced in CD4^+^ T cells and lower in those who die from sepsis compared to survivors (64). The STAT5 is also the key of aerobic glycolysis for naïve CD4^+^ T cells (65, 66) and the higher expression of glycolysis gene could be activated by STAT5 (67, 68). Glucose transporter 1 (GLUT1), a key protein for aerobic glycolysis, is decreased in CD4^+^ T cells of sepsis (69). The low levels of GLUT1 and aerobic glycolysis could be caused by the low STAT5 in sepsis (64). Mammalian target of rapamycin (mTOR) is also decreased in lymphocytes of sepsis patients (59, 70). Additionally, sepsis patients with multidrug-resistant bacterial infections exhibit lower counts of lymphocytes and monocytes than critically ill non-septic patients (71). CD4^+^ T cells are the key and IL-7 is essential for their differentiation (72), suggesting that higher IL-7 levels could promote more CD4^+^ T cells. However, the number of CD4^+^ T cells of sepsis or COVID-19 patients is often lower at 3–4 days after onset (56, 64). IL-7 can promote the function of naive CD4^+^FOXP3^+^ T cells, a subset of CD4^+^ T cells (73). The proportion of CD4^+^ FOXP3^+^ T cells is elevated in sepsis patients. An early rise in these cells among ICU patients is believed to have an adverse effect on sepsis outcomes (64, 74). CD25^+^ CD127^−^ T cells are detrimental to sepsis patients. In HIV patients, these cells can be reduced and restored by IL-7, whereas in sepsis patients, their levels tend to remain elevated (75–77). Toll-like receptor 5 (TLR5) can exacerbate sepsis and is decreased in monocytes of sepsis survivors (78, 79). In conclusion, the metabolism and proliferation of immune cells may be impaired, even in the presence of elevated IL-7 concentrations.

##### 3.1.3.2 The immune cells dysfunction

Human leukocyte antigen-DR (HLA-DR) serves as an indicator of immune system function. Its expression is reduced in monocytes and innate lymphoid cells (ILCs) of patients with sepsis and COVID-19, but increased in CD127^low^ PD-1^high^ T cells in sepsis (56, 64–66, 71, 80). Low programmed cell death 1 (PD-1) can mitigate sepsis-related damage. PD-1 is decreased in CD4^+^ T cells but remains elevated in CD8^+^ T cells. Meanwhile, programmed cell death ligand 1 (PD-L1) levels are maintained in monocytes of sepsis survivors (67, 68, 78, 79, 81). However, in sepsis patients with drug-resistant bacterial infections, PD-1 levels are increased in CD4^+^ and CD8^+^ T cells compared to critically ill non-septic patients. Similarly, PD-L1 levels are elevated in monocytes of these sepsis patients (71).

##### 3.1.3.3 Decompensated protective response

B- and T-lymphocyte attenuator (BTLA) plays a role in suppressing cytokine storms. Its expression is increased in CD4⁺ T cells of sepsis survivors (70, 82–84). Low interleukin-6 (IL-6) and high interleukin-10 (IL-10) levels are associated with improved outcomes in sepsis. However, both cytokines are frequently elevated in patients with sepsis (85–90), suggesting that the increase in IL-10 represents a compensatory response that is insufficient to prevent disease progression (65, 91, 92).

### 3.2 The mechanisms of exogenous IL-7 in sepsis

**Figure 2:**
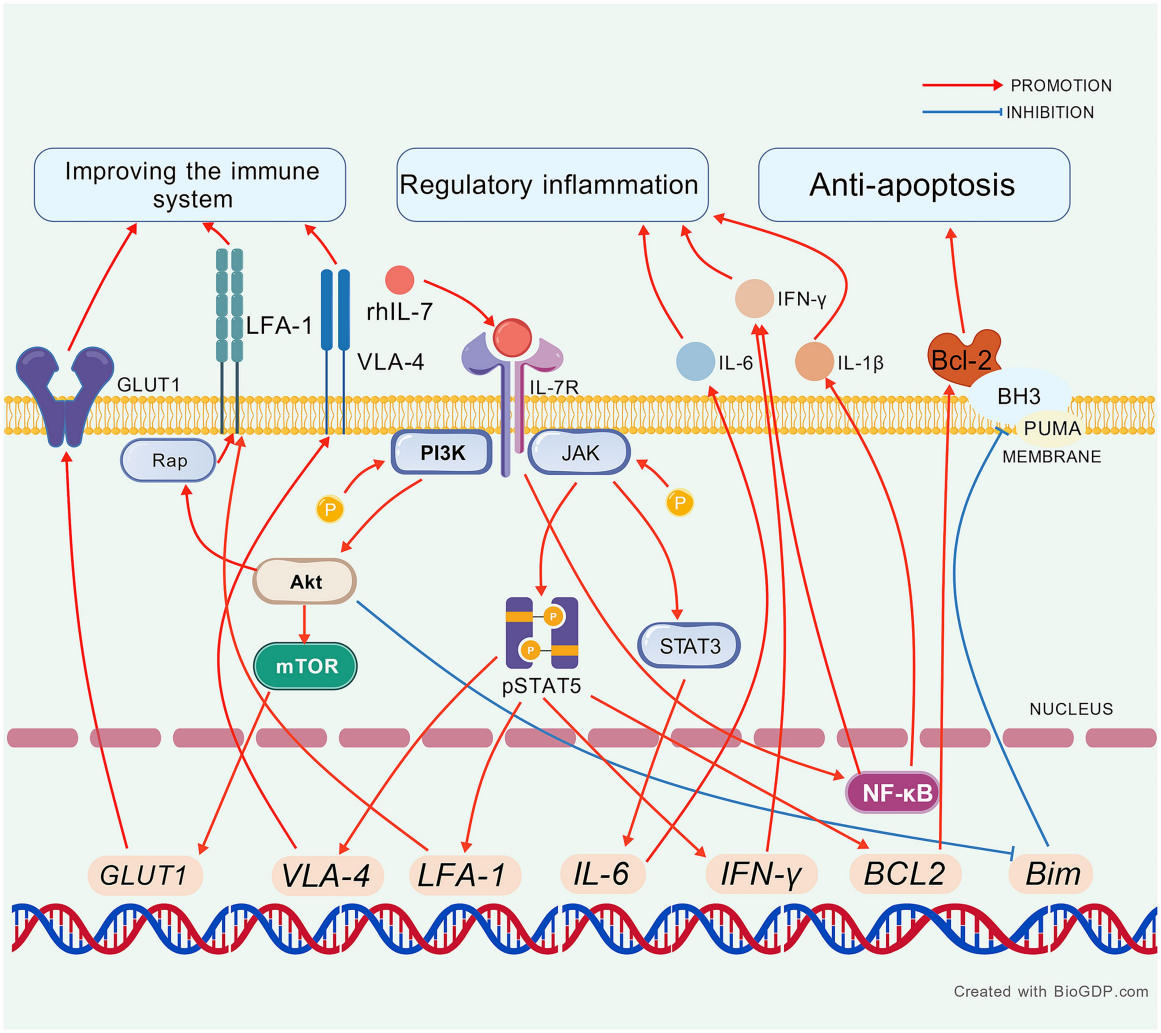
The mechanism of exogenous IL-7 in sepsis. Created with BioGDP.com (130). rhIL-7, recombinant human IL-7; LFA-1, lymphocyte function-associated antigen 1; VLA-4, very late antigen-4; IFN-γ, interferon-γ; NF-κB, nuclear factor-kappa B; Bcl-2, B-cell lymphoma-2; BH3, Bcl-2 binding components 3; PUMA, p53 up-regulated modulator of apoptosis; STAT5/3, signal transducers and activators of transcription 5/3; IL-6, interleukin-6; IL-1β, interleukin-1 beta; Rap-1, Ras-related protein 1.

We included 10 studies investigating the potential therapeutic mechanisms of exogenous IL-7 in sepsis (Table 2). Recombinant human IL-7 (rhIL-7) is widely used as a research agent. A newer type is the vaccinia virus Ankara (MVA)-human IL-7 (hIL-7)-Fc protein. This study builds on a 2021 systematic review that detailed the functions of IL-7 in sepsis and identified key molecular changes (93). The exogenous IL-7 to the bloodstream can help identify potential cures for immune system dysfunction and regulate inflammatory factors as well as inhibit apoptosis (Figure 2).

**Table 2:** The mechanism of exogenous IL-7 in sepsis.

References	Type	Dose	Model	Control	Target	Source of target	Change
(98)	rhIL-7	0.05 mg	Cecal ligation and puncture model of sepsis	Cecal ligation and puncture model of sepsis without treatment	CD4^+^ T cells, CD8^+^ T cells	Spleen and lymph nodes	Number of CD4^+^ T cells and CD8^+^ T cells ↑, Bcl-2 ↑, BH3 mRNA ↓, PUMA ↓, IL-7R ↓, LFA-1 ↑, VLA-4 ↑, memory T cells ↑
					Spleencytes	Spleen	IFN-γ ↑
(46)	rhIL-7	10 ng/mL	Cells from sepsis patients	Cells from sepsis patients without treatment	Lymphocytes	Plasma	Number of CD4^+^ T cells and CD8^+^ T cells ↑, IFN-γ ↑, Bcl-2 ↑, STAT5 ↑
(97)	rhIL-7	0.025 mg	Second-hit *C. albicans* model of sepsis	Second-hit *C. albicans* model of sepsis without treatment	CD4^+^ T cells, CD8^+^ T cells	Spleen	Number of CD4^+^ T cells and CD8^+^ T cells ↑, LFA-1 ↑, IFN-γ ↑, IL-6 ↑
(64)	rhIL-7	1 pg./mL	Cells from sepsis survivor	Cells from sepsis survivor without treatment	CD4^+^ T cells	Plasma	Number of CD4^+^ FOXP3^−^ T cells ↑, pSTAT5 ↑
	rhIL-7	10 pg/mL	Cells from sepsis survivor	Cells from sepsis survivor without treatment	CD4^+^ T cells	Plasma	Number of CD4^+^ FOXP3^−^ T cells ↑, pSTAT5 ↑
(57)	rhIL-7	100 ng/mL	Cells from sepsis patients	Cells from sepsis patients without treatment	T cells	Plasma	Number of CD8^+^ T cells ↑, mTOR ↑, Akt —, GLUT1 ↑
(96)	rhIL-7	0.025 mg	Second-hit *Pseudomonas aeruginosa* pneumonia model of sepsis	Second-hit *Pseudomonas aeruginosa* pneumonia model of sepsis without treatment	Lymphocytes	Lung and spleen	Number of lymphocytes ↑, function of T cells secreting cytokines ↑
					Lungcytes	Lung	NF-kB ↑, STAT3 ↑
(71)	rhIL-7	50 ng/mL	Sepsis patients of multidrug resistant bacterial	Critically-ill non-septic patients	Lymphocytes	Plasma	IFN-γ —
(95)	rhIL-7	0.025 mg	Cecal ligation and puncture model after sepsis	Cecal ligation and puncture model after sepsis without treatment	CD8^+^ T cells	Spleen	Number of CD8^+^ T cells ↑
(94)	MVA-hIL-7-Fc	10^7^–10^8^ pfu or 5 μg	Cecal ligation and puncture model of sepsis	Cecal ligation and puncture model of sepsis by rhIL-7-Fc	CD4^+^ T cells, CD8^+^ T cells	Lung and spleen	Number of CD4^+^ T cells and CD8^+^ T cells ↑, Bcl-2 ↑, IFN-γ ↑, IL-1β ↑
(56)	MVA-hIL-7-Fc	100 ng/mL	Cells from sepsis patients and COVID-19 patients	Baseline	CD3^+^ T cells	Plasma	pSTAT5 ↑
	MVA-hIL-7-Fc	100 ng/mL	Cells from sepsis patients and COVID-19 patients	Baseline	T cells	Plasma	Number of lymphocytes ↑, function of T cells secreting cytokines ↑

IL-7R, IL-7 receptor; rhIL-7, recombinant human IL-7; MVA, vaccinia virus Ankara; LFA-1, lymphocyte function-associated antigen 1; VLA-4, very late antigen-4; IFN-γ, interferon-γ; NF-κB, nuclear factor-kappa B; Bcl-2, B-cell lymphoma-2; BH3, Bcl-2 binding components 3; PUMA, p53 up-regulated modulator of apoptosis; Akt, protein kinase B; STAT5, signal transducers and activators of transcription 5; IL-6, interleukin-6; IL-1β, interleukin-1 beta; ↑, increase; ↓, decrease; —, maintain.

#### 3.2.1 Improving the immune system

Several studies have reported increased lymphocyte counts in the spleen and lymph nodes of mouse models (94–98), with corresponding increases in CD4⁺ and CD8⁺ T-cell subsets. *In vitro* studies using plasma from patients with sepsis have demonstrated an increase in lymphocytes following treatment with rhIL-7 (46, 56, 57, 64). Memory T cells are vital T cell subsets, and rhIL-7 can aid in their preservation in sepsis (98). Lymphocyte function-associated antigen 1 (LFA-1) is leukocyte adhesion marker involved in cell migration also very late antigen-4 (VLA-4). In mice, these markers can be enhanced by rhIL-7 via protein kinase B (Akt)/Ras-related protein 1 (Rap-1) and STAT5 (97–100). The rhIL-7 not only increases the number and migratory capacity of lymphocytes but also enhances their aerobic glycolysis and secretory functions, in both animal models and human blood (46, 56, 57, 96, 97). Overall, the therapeutic use of rhIL-7 shows great promise in combating sepsis-induced lymphopenia and immune dysfunction.

#### 3.2.2 Regulatory inflammation

Interferon-*γ* (IFN-γ) has an important function for activating the cellular immunity and IL-7 could induce it to regulate the immune system (101–104). IFN-γ was increased in spleen of sepsis mice also in blood of sepsis patients, survivors, and COVID-19 patients by rhIL-7 and MVA-hIL-7-Fc. However, the increased IFN-γ in sepsis is same compared the critically-ill non-septic patients (46, 71, 94, 96, 97). The nuclear factor-kappa B (NF-κB) interacts with IL-7 and promote the inflammation, could be increased in sepsis mice by rhIL-7 (96, 105–108). IL-6 is the dependent cytokine for the viral clearance of IL-7, which could be increased in spleen of sepsis mice by rhIL-7 (97, 109). The increased interleukin-1 beta (IL-1β) and IL-6 could be found in spleen and lung of sepsis mice and the higher levels could be seen by MVA-hIL-7-Fc versus by rhIL-7, which could regulate the function of IL-7 (94, 110, 111).

#### 3.2.3 Anti apoptosis

IL-7 promotes thymocyte proliferation via B-cell lymphoma-2 (Bcl-2) in T cells derived from septic animals and human sources. MVA-hIL-7-Fc can induce higher Bcl-2 levels than rhIL-7 in the lungs and spleens of sepsis mice (46, 94, 98). Bcl-2 binding components 3 (BH3) and p53 up-regulated modulator of apoptosis (PUMA) are pro-apoptotic factors in sepsis. These factors can be decreased by rhIL-7 in mouse models (98, 112, 113). STAT3 and STAT5 are key pathways involved in apoptosis. Both rhIL-7 and MVA-hIL-7-Fc can increase these pathways in sepsis mice or the blood of sepsis patients (46, 56, 96). According to the above text, the STAT5 also is the key of glycolysis and histone lactylation (63, 114–116) and the change of immune function could attribute to the higher STAT5. The mTOR is another pathway involved in apoptosis via IL-7. It is increased in the blood of sepsis patients, while Akt levels remain unchanged (57, 117). In short, IL-7 therapies combat sepsis-induced lymphocyte apoptosis and metabolic dysfunction by modulating key survival and signaling pathways.

### 3.3 The clinical application of IL-7 in sepsis

We included nine studies investigating the clinical application of IL-7 in sepsis (Tables 3, 4). We found that endogenous IL-7 in the blood may serve as a marker for assessing disease severity and prognosis in elderly patients with sepsis. Exogenous IL-7, such as rhIL-7, may represent a potential immunotherapeutic agent.

**Table 3:** The IL-7 as the prognosis marker of sepsis.

References	Type	Duration	Experiment	Control	Source	Trend	Mortality
(118)	IL-7	NA	Sepsis shock	Severe sepsis	Plasma	↑	NA
	IL-7	NA	Sepsis survivor	Sepsis death	Plasma	NA	NA
(45)	sIL-7R	1, 3 days	Sepsis survivor	Sepsis death	Plasma	↑	+
	IL-7R mRNA	3 days	Sepsis survivor	Sepsis death	Plasma	↑	−
(119)	IL-7	1, 3, 5 days	Sepsis shock	Severe sepsis	Serum	NA	NA
	IL-7	1, 3, 5 days	Sepsis survivor	Sepsis death	Serum	↑	NA
(122)	*IL7R*	1 days	Sepsis patients	Health	Plasma	↓	NA
(120)	IL-7	1 days	Sepsis elderly survivor	Sepsis elderly death	Plasma	↓	+
(121)	IL-7	1, 5 days	Sepsis patients (5 days)	Sepsis patients (1 days)	Plasma	↓	+

sIL-7R, soluble IL-7R; +, positive; −, negative; ↑, increase; ↓, decrease.

**Table 4:** The IL-7 as a therapeutic agent of sepsis.

References	Type	Dose	Frequency	Duration	Method	Patients	Control	Function	Mortality	Adverse effect	Safety
(85)	rhIL-7	10 μg/kg	Twice in first week, once/week	4 weeks	Intramuscular injection	Sepsis patients with treatment	Placebo	TNF-α and IL-6 —, IL-10 ↑, number of lymphocytes ↑, number of CD4^+^ T cells ↓ then ↑, percentage of Ki67 positive CD4^+^ T cells ↑, percentage of Ki67 positive CD8^+^ T cells ↑, percentage of IL-7R positive CD4^+^ T cells and CD8^+^ T cells ↓, percentage of CD38 positive CD4^+^ T cells ↑	Maintain	Rashes	Safe
	rhIL-7	10 μg/kg	Twice/week	4 weeks	Intramuscular injection	Sepsis patients with treatment	placebo	TNF-α and IL-6 —, IL-10 ↑, number of lymphocytes ↑, number of CD4^+^ T cells and CD8^+^ T cells ↓ then ↑, number of neutrophils ↑, percentage of Ki67 positive CD8^+^ T cells ↑, percentage of Ki67 positive CD8^+^ T cells ↑, percentage of CD38 positive CD4^+^ T cells ↑	Maintain	Rashes	Safe
(123)	rhIL-7	10 μg/kg	Twice or three times/weeks	90 days	Intravenous injection	Sepsis patients with treatment	Placebo	Number of lymphocytes ↑, number of CD4^+^ T cells and CD8^+^ T ↑, IL-6, IL-10, and TNF-α —	Maintain	Fever, respiratory distress	Safe
(41)	rhIL-7	10 μg/kg	twice/week	3–4 weeks	intramuscular injection	sepsis patients of COVID-19 with treatment	placebo	TNF-α, IL-6, and IL-10 —, ICU length ↓, secondary infection ↓, hospital length ↓	maintain	NA	safe

IL-7R, IL-7 receptor; rhIL-7, recombinant human IL-7; IL-6, interleukin-6; IL-10, interleukin-10; TNF-α, tumor necrosis factor-α; HLA-DR, human leukocyte antigen DR; PD-1, programmed cell death 1; ↑, increase; ↓, decrease; —, maintain.

#### 3.3.1 As the prognosis marker of sepsis

Endogenous IL-7 levels vary between septic shock and severe sepsis patients, yet no significant difference is observed between sepsis survivors and non-survivors in plasma (118). One study revealed a change in serum IL-7 between sepsis survivors and non-survivors, but it cannot be regarded as a mortality marker (119). Interestingly, we found that endogenous IL-7 levels differ between elderly sepsis survivors and elderly sepsis deaths, with higher endogenous IL-7 associated with higher mortality (120). Among patients with sepsis, lower plasma levels of endogenous IL-7 may be associated with increased mortality over time (121). Bioinformatics analysis suggests that IL-7R may also serve as an underlying marker for sepsis (122). Soluble IL-7R (sIL-7R) levels differ between sepsis survivors and sepsis deaths in plasma, with higher sIL-7R associated with higher mortality. In contrast, IL-7 mRNA levels in plasma have an inverse relationship with mortality (45). Overall, endogenous IL-7 may serve as a prognostic marker in elderly patients with sepsis, whereas sIL-7R may be used to evaluate prognosis in the general sepsis population. Reduced IL-7 mRNA levels inversely correlate with survival, hinting at transcriptional suppression in fatal cases.

#### 3.3.2 As the immunotherapy agent in sepsis

The typical dose of rhIL-7 is 10 μg/kg, administered once or twice weekly. Francois et al. (85) reported that intramuscular administration of rhIL-7 in patients with sepsis increased the number of lymphocytes and their subsets, as well as IL-10 levels; however, mortality remained unchanged, suggesting that immune recovery alone may be insufficient to reverse severe infection and organ damage. The only adverse effect observed was rashes, which resolved after discontinuing rhIL-7, indicating that intramuscular injection of rhIL-7 is safe. Intravenous administration of rhIL-7 results in higher blood concentrations compared to intramuscular injection. Patients receiving intravenous rhIL-7 experienced increased lymphocyte counts without cytokine storms. However, fever and respiratory distress may occur as potential side effects (123). Shankar-Hari et al. (41) reported that COVID-19 sepsis patients treated with rhIL-7 had shorter ICU and hospital stays and a lower risk of secondary infections, though mortality rates were unchanged. Administered at standard doses (10 μg/kg, weekly or biweekly) via intramuscular or intravenous routes, rhIL-7 safely increases lymphocyte subsets including CD4^+^/8^+^ T cells and modulates cytokines like IL-10, with manageable side effects like transient rashes and fever. However, clinical trials, including those involving COVID-19-associated sepsis, have not demonstrated a reduction in mortality, despite shorter ICU and hospital stays and a lower incidence of secondary infections.

## 4 Discussion

Sepsis is a major contributor to patient mortality and has diverse causes. Its hallmark is infection-induced organ dysfunction, and it imposes a substantial socioeconomic burden. Current treatments are limited, primarily targeting bacteria or viruses, which can lead to drug resistance and the emergence of superbugs. Recently, immunotherapy has gained attention, with PD-L1, IL-7, and others emerging as key therapeutic candidates (124). This article reviews the changes and effects of both endogenous and exogenous IL-7 in sepsis.

We try to demonstrate the continuous progression of changes in endogenous IL-7 and related factors based on the above results. In sepsis, blood levels of endogenous IL-7 are typically elevated initially as a result of immune cell activation and endothelial damage caused by the infection. This initial response is part of immune system. However, the subsequent cytokine storm and pathogen-induced damage rapidly deplete normal lymphocytes, leading to a decline in immune function. In response to this overwhelming immune activation, regulatory mechanisms are triggered. The percentage of CD4^+^ FOXP3^+^ and CD25^+^ CD127^−^ T cells increased to suppress cytokine storm, as evidenced by elevated BTLA levels. However, this regulatory response is often insufficient to control the pathogen load, leading to further lymphocyte depletion. Elevated IL-6 from the cytokine storm exacerbates endothelial damage, resulting in increased IL-7 release. With the release of IL-7, expression of the IL-7R increases. Meanwhile, damage to endothelial cells results in reduced IL-7 mRNA levels. Despite elevated IL-7 levels, the capacity of immune cells to perform essential functions is impaired. The rapid turnover of immune cells results in defective glycolysis, as evidenced by reduced STAT5, GLUT1, and HLA-DR expression. Proliferation is further inhibited by increased PD-L1 and diminished mTOR activity. Activation of PD-L1 may be initiated by STAT5 through the facilitation of histone lactylation during immune suppression (78), suggesting that the elevated PD-L1 observed is pathogen-induced, in line with the above findings. While IL-10 may offer some support to IL-7, its overall impact is limited by the severely compromised immune system. Additionally, low TLR5 levels indicate reduced pathogen-killing capacity. Ultimately, the combination of immune dysfunction and endothelial changes leads to a persistent and stable state of immune suppression. Elevated IL-7 levels are maintained due to reduced clearance mechanisms, further complicating the recovery process. Collectively, dysregulated IL-7 is closely linked to sepsis pathology, immune compensation, and clinical outcomes. Its sustained elevation may reflect the body’s attempt to restore immune homeostasis, and targeted modulation of this pathway could offer therapeutic opportunities to improve sepsis management and survival.

The primary mechanisms of IL-7 *in vitro* for sepsis animal and human blood include immune system recovery, inflammation regulation, and anti-apoptosis. A network can be illustrated where rhIL-7 enters the bloodstream and upregulates STAT5/3 and GLUT1 to enhance glycolysis. rhIL-7 also modulates apoptosis-related factors: it increases Bcl-2 while decreasing BH3 and PUMA, and it elevates mTOR levels, all of which contribute to reducing immune system depletion. Additionally, rhIL-7 upregulates LFA-1 and VLA-4, promoting the migration of white cells and thereby replenishing the immune’s resources. In terms of inflammation, rhIL-7 increases the levels of inflammatory factors like IFN-*γ*, IL-6, NF-κB, and IL-1β to combat pathogens. Notably, NF-κB can promote IL-7 production, thereby enhancing the efficacy of rhIL-7. Ultimately, these mechanisms work together to restore the immune system and reduce pathogen load.

In clinical settings, endogenous IL-7 and IL-7R have shown promise as prognostic markers in sepsis. These markers may offer valuable insights into the mortality risk in sepsis. The above findings highlight the complexity of IL-7 biology in sepsis, with its prognostic value influenced by age, anatomical compartment, and molecular form. Clinically, integrating IL-7, sIL-7R, and IL-7 mRNA measurements could enhance risk stratification; however, their interpretation requires careful contextualization to avoid paradoxical conclusions. Future studies should validate these markers in stratified cohorts and investigate the mechanisms underlying their discordant associations with clinical outcomes.

Moreover, rhIL-7 has demonstrated considerable potential in boosting the immune system of sepsis patients. This enhancement can lead to shorter hospital stays and a reduced likelihood of developing secondary infections, both of which are critical factors in improving patient outcomes. The high safety profile of rhIL-7 is another major advantage, making it an attractive therapeutic option for managing sepsis. Given the complexity and severity of sepsis, a safe and effective treatment that can enhance the immune system and reduce complication risks represents a significant advancement in managing this condition. However, exogenous IL-7 has the potential to reverse immune dysfunction in patients with sepsis, although mortality rates remain unchanged. This suggests that, while IL-7 may enhance immune function, it does not address the broader pathophysiology of sepsis. The discrepancy between improved immune parameters and unaltered survival underscores the need for precision medicine approaches to optimize IL-7 therapy. Precision medicine, characterized by tailoring treatment to the individual through artificial intelligence and large-scale modelling, has already been applied in oncology (124). Genomic, microbic, and radiomic analyses are key methods for detecting differences (76, 125–127). The principles of precision medicine may be applied to the clinical use of IL-7, given the variability in IL-7 mRNA and IL-7R mRNA expression. Precision medicine strategies should be considered in future clinical trials of IL-7. The discrepancy between improved immune parameters and unchanged survival underscores the need to optimize IL-7 therapy through tailored approaches. Artificial intelligence-driven multi-omics methods, including genomics, microbiome profiling, and radiomics, could facilitate the stratification of patients with sepsis according to IL-7/IL-7R mRNA variability, microbial ecology, or metabolic–epigenetic states. For example, selecting patients with low endogenous IL-7, specific IL-7R polymorphisms, or suppressed STAT5-glycolytic pathways may enhance therapeutic efficacy. Likewise, biomarker-guided dosing based on sIL-7R levels or lymphocyte recovery kinetics could mitigate the risk of hyperinflammation or futile immune activation. Combining IL-7 with adjunctive therapies and employing precision biomarkers could unlock its full potential to transform sepsis management beyond immune reconstitution.

However, several limitations should be noted: variability in patient responses, lack of FDA approval, and small trial sizes, all of which reduce the reliability of the findings. Employing endogenous IL-7 and IL-7R as prognostic markers in sepsis currently lacks a well-defined standard. Given the intricate and variable nature of sepsis, depending solely on IL-7 or IL-7R may not adequately reflect the disease’s multifactorial nature. Integrating IL-7 with additional markers, such as PD-L1, into a multi-marker strategy could yield a more robust and nuanced assessment of patient conditions. This comprehensive approach may enhance diagnostic accuracy, refine risk stratification, and offer deeper insights into disease trajectories and therapeutic efficacy (76, 118, 119, 121, 122, 125, 126). A comprehensive and systematic evaluation plan that integrates IL-7 with other relevant markers is valuable for advancing our idea and clinical management of sepsis. Most existing studies have focused on *in vitro* experiments, which, though valuable, do not fully translate to clinical settings. As a result, determining the optimal clinical dosing regimen for rhIL-7 remains a critical area for further investigation. Additionally, the potential synergistic effects of combining rhIL-7 with conventional treatments such as antibiotics or antivirals need to be rigorously tested through experimental studies to validate their efficacy and safety in clinical practice. Moreover, the choice of administration method is pivotal. Both intravenous and intramuscular injection routes need to be thoroughly evaluated to determine which method offers the best balance of efficacy, safety, and patient tolerance. The formulation of the drug itself is another crucial factor that can significantly impact its therapeutic effectiveness. For instance, the MVA-hIL-7-Fc fusion protein has demonstrated superior outcomes in preclinical mouse models compared to other forms of IL-7, highlighting the importance of exploring innovative dosage forms to enhance drug performance (56, 94, 127, 128). In addition to the points discussed earlier, precision medicine could be leveraged based on the specific genetic profiles of patients. Endothelial function and immune cell counts are crucial in sepsis pathophysiology and could guide personalized treatments. Therefore, collecting individual data on blood and vascular health could guide the targeted use of rhIL-7. Regarding the dosing of rhIL-7, it would be rational to administer it at a concentration sufficient to elevate IL-7 levels above the median observed in sepsis survivors. This approach aligns with the underlying mechanisms of IL-7’s action. Additionally, considering the complex interactions among cytokines, such as the upregulation of IL-7 by NF-κB, could further refine treatment protocols. It should be noted that endothelial damage is a major factor in sepsis. Although rhIL-7 may not reverse this damage, it could potentially worsen it. Therefore, adjunctive therapies aimed at promoting endothelial repair, such as PCSK9 inhibitors, should be considered to mitigate these effects (129). Additionally, it is important to consider that STAT5 may promote the PD-L1 in white cells through histone lactylation. This suggests that PD-1 inhibitors could be beneficial in modulating this pathway and enhancing the therapeutic effects of IL-7. Further research is essential to explore these areas more thoroughly.

## 5 Conclusion

In summary, endogenous IL-7 and IL-7R levels can be elevated in sepsis; however, this does not reverse the dysfunction and depletion of immune cells, even when accompanied by other decompensatory changes. These findings indicate that endogenous IL-7 and IL-7R have potential as prognostic markers in sepsis. Exogenous IL-7 can enhance immune function, regulate inflammation, and exert anti-apoptotic effects, but it does not reduce sepsis-related mortality, suggesting that its therapeutic potential may require combination with other interventions. Further research is needed to elucidate the complex interactions between IL-7 and other immune components, as well as its role in modulating inflammation and promoting immune recovery in sepsis. More comprehensive and rigorous clinical trials will be essential to optimize IL-7-based therapy and improve outcomes in this condition.
